# Imaging features of hepatic sinusoidal obstruction syndrome or veno-occlusive disease in children

**DOI:** 10.1007/s00247-021-05174-w

**Published:** 2021-11-03

**Authors:** Anneloes E. Bohte, Miranda P. Dierselhuis, Max M. van Noesel, Maarten H. Lequin

**Affiliations:** 1 Princess Máxima Center for Pediatric Oncology, Heidelberglaan 25, 3584 CS, Utrecht, The Netherlands; 2 Department of Radiology and Nuclear Medicine, University Medical Center Utrecht/Wilhelmina Children’s Hospital, Utrecht, The Netherlands

**Keywords:** Children, Computed tomography, Doppler ultrasound, Liver, Magnetic resonance imaging, Oncology, Sinusoidal obstruction syndrome, Ultrasound, Veno-occlusive disease

## Abstract

Hepatic sinusoidal obstruction syndrome, also known as veno-occlusive disease, can occur as a complication of myeloablative chemotherapy, as a result of low-intensity chemotherapy-related liver toxicity or radiotherapy of the liver. Symptoms of sinusoidal obstruction syndrome can range from asymptomatic to liver dysfunction or severe disease with life-threatening acute multi-organ failure. Imaging features can suggest or support this clinical diagnosis. Familiarity with the imaging spectrum of sinusoidal obstruction syndrome is therefore important for both radiologists and clinical oncologists. Here, multi-modality radiologic appearances of sinusoidal obstruction syndrome in pediatric patients are illustrated, including outcome after follow-up.

## Introduction

Hepatic sinusoidal obstruction syndrome, also known as veno-occlusive disease, reflects the cascade caused by injury to sinusoidal endothelial cells and hepatocytes, leading to non-thrombotic obstruction of centrilobular veins by necrosis and detachment of subendothelial connective tissue [1, 2]*.* Triggers for sinusoidal obstruction syndrome include drug toxicity, the release of cytokines and inflammation, alloreactivity in hematopoietic stem cell transplantation and protein C anticoagulant pathway abnormalities. In reaction to the sustained damage, sinusoidal fibrosis and narrowing of central veins can occur*.* Consequently, impairment of liver blood inflow and outflow, as well as signs and symptoms comparable to portal hypertension and its sequelae, can develop [3, 4].

In children, sinusoidal obstruction syndrome is most frequently seen in relation to the myeloablative conditioning regimen in allogeneic hematopoietic stem cell transplantation or autologous stem cell transplantation with an incidence of approximately 20–60% [5]. It is less frequently seen as a complication of actinomycin-D-containing chemotherapy regimens, administered in nephroblastoma, rhabdomyosarcoma, Ewing sarcoma and certain brain tumors [1, 6, 7].

Risk factors for developing sinusoidal obstruction syndrome are younger age, higher chemotherapy dose, previous radiotherapy and lower body weight*.* In nephroblastoma, right-side tumor location is an additional risk factor, possibly due to .mechanical compression of the liver [8]*.*

Patients with sinusoidal obstruction syndrome typically present with right upper quadrant pain, hepatomegaly, jaundice, ascites and weight gain secondary to fluid retention. However, symptoms can range from asymptomatic to encephalopathy or acute multi-organ failure. The clinical course is often self-limiting, although mortality rates can be high in severe cases with multi-organ failure [1].

Since the 1980s, the Seattle or Baltimore criteria have been used to diagnose sinusoidal obstruction syndrome in both adults and children. Both scoring systems include elevated bilirubin levels, hepatomegaly and weight gain within 21 days of hematopoietic stem cell transplantation. In addition, the Baltimore criteria also include ascites [9, 10]. Recently, the European Society for Blood and Marrow Transplantation has proposed new diagnostic and severity criteria for pediatric sinusoidal obstruction syndrome, in which the time limitation for the onset of sinusoidal obstruction syndrome has been omitted and consumptive and transfusion refractory thrombocytopenia has been included (Table 1) [1]. The availability of effective treatment of sinusoidal obstruction syndrome by defibrotide prompts a timely diagnosis of severe sinusoidal obstruction syndrome, since early initiation of defibrotide is associated with higher survival [11–13]. The main role of imaging is to either confirm the diagnosis of suspected sinusoidal obstruction syndrome or to differentiate between other causes of liver damage that would require different treatment strategies (e.g., graft-versus-host-disease, cholestasis or cholangitis, mycotic infections, viral hepatitis, Budd-Chiari, medication-induced hepatitis or acute heart failure). There is additional potential for imaging with respect to early diagnosis of sinusoidal obstruction syndrome, which could directly influence patient outcome. Hence, familiarity with the various imaging features is crucial for both (pediatric) radiologists and oncologists. Since ultrasound (US) is the main screening modality for sinusoidal obstruction syndrome, most studies report on US-based imaging features, and less so for computed tomography (CT) and magnetic resonance imaging (MRI) [14]. However, an illustrated overview of the different imaging appearances of sinusoidal obstruction syndrome including its course over time is lacking. We therefore present this pictorial essay on pediatric sinusoidal obstruction syndrome with recommendations for radiologic follow-up.

**Table 1:** European Society for Blood and Marrow Transplantation diagnostic criteria for hepatic sinusoidal obstruction syndrome in children (adapted from [1])

No limitation for time of onset of sinusoidal obstruction syndrome	
The presence of two or more of the following:	
• Unexplained consumptive and transfusion-refractory thrombocytopenia	
• Otherwise unexplained weight gain on 3 consecutive days despite the use of diuretics, or a weight gain >5% above baseline	
• Hepatomegaly (best confirmed by imaging) above baseline value	
• Ascites (best confirmed by imaging) above baseline value	
• Rising bilirubin from a baseline value on 3 consecutive days or bilirubin ≥2 mg/dL within 72 h	

## Ultrasonography

Ultrasonography allows for the examination of the liver and abdomen in several ways, including conventional high-resolution B-mode, color Doppler and spectral Doppler studies, elastography and contrast-enhanced US.

### B-mode and Doppler US

In the literature, conventional (B-mode) US characteristics of sinusoidal obstruction syndrome include hepato(spleno)megaly, ascites, periportal and gallbladder wall edema, increased width of the portal vein, and indistinct borders or narrowing of hepatic veins. Doppler-mode characteristics include portal vein flow demodulation, decreased flow velocity or reversal of portal vein flow, decreased spectral density, increased resistive index and peak systolic velocity of the hepatic artery, congestion index, monophasic reduced flow in the hepatic veins, and visualization of collateral veins (e.g., in the paraumbilical veins)*.* These characteristics are summarized in Table 2 [1, 5, 15–20]. For the Doppler examination, a right intercostal approach is preferred because the Doppler angles for performing velocity measurements and evaluation of flow are optimal. Furthermore, the liver can also be evaluated at the parenchymal segmental level without exercising probe pressure onto the liver, and thereby interacting with sensitive portal venous inflow at this level [21]*.*

**Table 2:** Literature overview of studied US parameters for sinusoidal obstruction syndrome in children

B-mode	*Definition*	Doppler	*Definition*	Elastography	*definition*
Hepatomegaly	*>1-cm increase in size relative to baseline measurement* [5, 15, 16] *More than 2 SD above normal for age* [5]	Demodulation of portal vein flow	*Disappearance of velocity variations with breathing* [15]	Increased liver stiffness	*Cutoff for increased liver stiffness is vendor and technique dependent*
Splenomegaly	*>1-cm increase in size relative to baseline measurement* [15]	Decrease in spectral density of portal vein flow	*Decline in the amount of red cells in portal flow* [15]		
Periportal edema	*–*	Portal vein flow	*Decrease (<10 cm/s) or reversal of portal vein flow* [15, 16]		
Gallbladder wall edema	*>4 mm* [16, 17] *>6 mm* [15] *>10 mm* [18]	Increased resistive index of the hepatic artery	*>0.8 * [19] *>0.75* [15, 18] *>0.70* [16]		
Hepatic vein narrowing	*<3 mm, measured at 2 cm from the inferior vena cava* [15]	Hepatic artery peak systolic velocity	*Common hepatic artery:* *31 cm/s and 123 cm/s cutoffs for 78% sensitivity and 54% specificity in higher risk and lower risk groups, respectively* ^*a,b*^ *Left hepatic artery:* *35 cm/s and 83 cm/s cutoffs for 74% sensitivity and 70% specificity in higher risk and lower risk groups, respectively* [20] ^*a,b*^		
Increased portal vein diameter	*>8 mm* [15, 16]	Congestion index	*Cross-section area of portal vein divided by average blood flow velocity, <0.1* [15]		
Indistinct hepatic vein borders	*–*	Hepatic vein flow	*Reduced, monophasic flow in right hepatic vein* [17] *measured >8 cm from inferior vena cava* [19]		
Ascites	*Minimal, moderate or need for external drainage* [1] *Mild: minimal fluid by liver, spleen or pelvis* *Moderate: <1 cm fluid.* *Severe: fluid in all three regions, >1 cm in ≥2 regions* [5]	Visualization of collateral veins	*E.g., in the paraumbilical veins, seen as a hypoechoic lumen in the hyperechoic round ligament* [2, 15, 17, 19]		

^a^Higher risk group: busulfan conditioning regimen before hematopoietic stem cell transplantation (HSCT)

^b^Lower risk group: no busulfan conditioning regimen before HSCT

*SD* standard deviation

### Elastography

Limited data are available on US-based liver stiffness measurements in pediatric sinusoidal obstruction syndrome. One of the first publications dates from 2016, and results are encouraging [4, 14]. An increase of liver stiffness from baseline correlates with the onset of sinusoidal obstruction syndrome after hematopoietic stem cell transplantation, with stiffness values decreasing as the condition resolves [22]*.* Liver stiffness has been shown to increase even before other imaging and clinical findings of sinusoidal obstruction syndrome become apparent [4, 14]*.* Corbacioglu et al. [12] showed that an earlier start of defibrotide treatment of sinusoidal obstruction syndrome was associated with a more favorable outcome in children undergoing hematopoietic stem cell transplantation. Therefore, structural liver stiffness measurements including baseline measurements are now increasingly advocated in patients at risk of developing sinusoidal obstruction syndrome in order to stratify those who could benefit from early defibrotide treatment [14]*.* Most recently, Chan et al. [23] have summarized the available literature on US elastography in sinusoidal obstruction syndrome.

### Contrast-enhanced US

Recently, Trenker et al. [24] have shown in adults undergoing allogeneic stem cell transplantation that there may be a role for contrast-enhanced US in detecting preclinical sinusoidal obstruction syndrome by means of hypo-enhancement of the liver parenchyma. To our knowledge, no data in children are available yet.

**Fig. 1:**
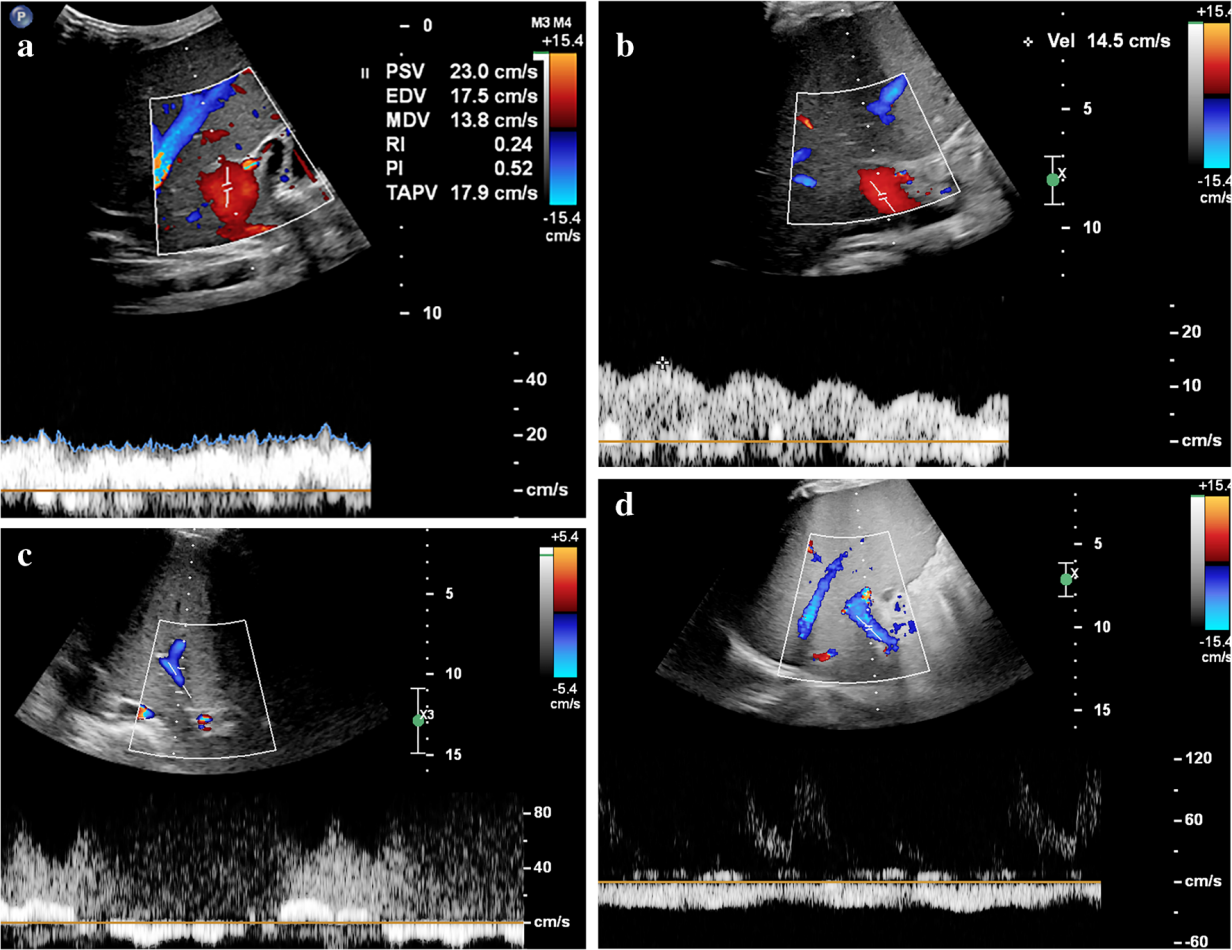
Different hepatic portal vein flow patterns can be observed in sinusoidal obstruction syndrome. **a** An axial intercostal transverse color Doppler ultrasound (US) image of the liver shows normal hepatic portal flow direction, velocity and pattern in a 10-month-old boy with juvenile myelomonocytic leukemia who developed severe sinusoidal obstruction syndrome 7 days after allogeneic stem cell transplantation. This was the initial US examination; hepatomegaly and ascites confirmed the clinical suspicion of sinusoidal obstruction syndrome. **b** An axial intercostal transverse color Doppler US image of the liver in a 4-year old girl with severe sinusoidal obstruction syndrome 21 days after autologous stem cell transplantation, and 5 days after the first clinical suspicion based on elevation of liver transaminases. Increased pulsatility of the portal vein flow pattern with decreased velocities of 10–15 cm/s. **c** An axial intercostal transverse color Doppler US image of the liver in a 14-year-old girl with neuroblastoma and severe sinusoidal obstruction syndrome shows a to-and-fro flow pattern 5 weeks after autologous stem cell transplantation and 1 week after the first clinical suspicion of the disease. **d** An axial intercostal transverse color Doppler US image of the liver in a 4-year-old boy with severe sinusoidal obstruction syndrome 29 days after autologous stem cell transplantation shows hepatofugal flow direction in the portal vein. Abnormal portal vein flow patterns resolved on follow-up, in keeping with the clinical resolution of sinusoidal obstruction syndrome

**Fig. 2:**
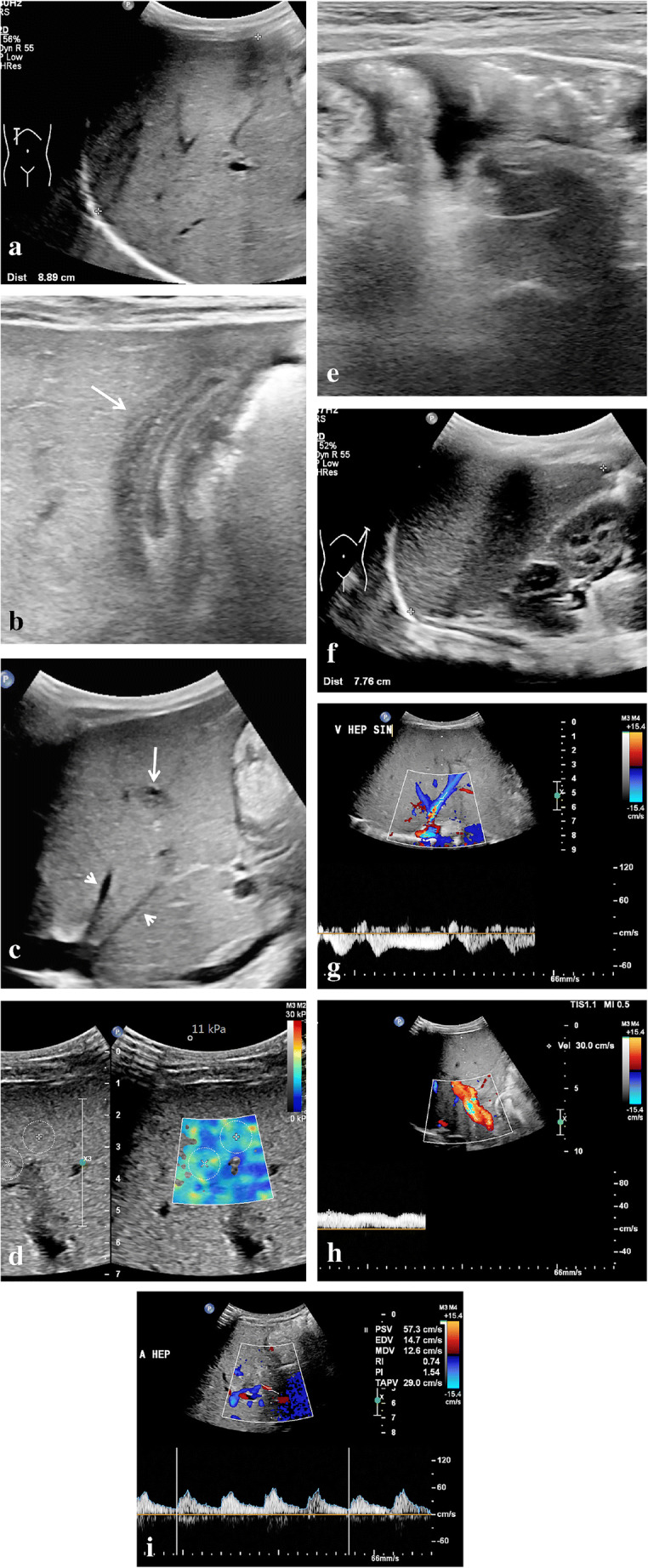
Ultrasound (US) characteristics of mild sinusoidal obstruction syndrome in a 9-month-old boy with right-side nephroblastoma who completed the first month of postoperative treatment with vincristine and actinomycin D. He presented with thrombocytopenia and elevated aspartate transaminase (2-5x above upper limit). A US examination showed several signs complementary to the clinical suspicion of sinusoidal obstruction syndrome. **a** A sagittal US image shows an enlarged age-related liver size of 8.9 cm. **b** A transverse high-resolution US image shows gallbladder wall edema (*arrow*). **c** A sagittal US image of the liver shows periportal edema (*long arrow*) and narrowing of the hepatic veins (*short arrows*). **d** An axial intercostal elastography measurement of the right liver lobe shows elevated stiffness of 11 kPa (normal stiffness: 5–6 kPa). **e** A transverse high-resolution US image of the lower abdomen shows mild to moderate ascites. **f** A sagittal US image of the spleen shows an age-related enlarged spleen of 7.8 cm. **g** A Doppler US of the liver shows preserved hepatic vein flow pattern. **h** A Doppler US of the liver shows preserved portal vein flow direction with increased velocity (30 cm/s). **i** A Doppler US of the liver shows a borderline hepatic artery resistive index of 0.74. Based on the predicted mild to moderate sinusoidal obstruction syndrome, this patient was treated with thrombocyte-transfusion and fluid restriction. The condition of the patient including laboratory values soon improved

**Fig. 3:**
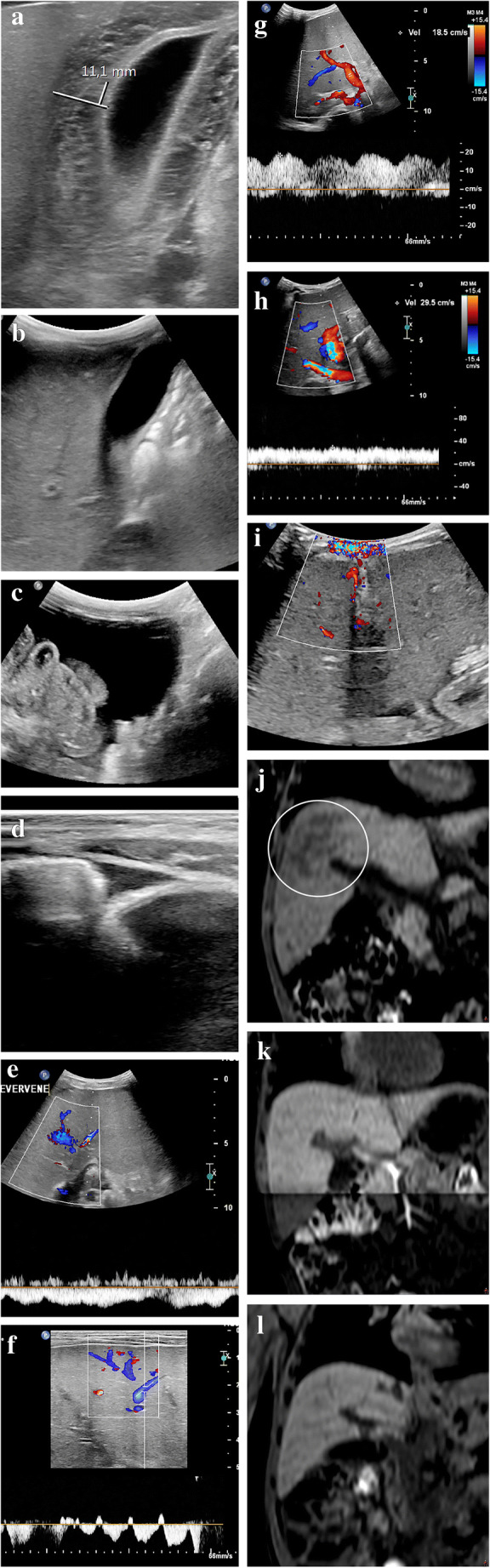
Ultrasound (US) findings of severe sinusoidal obstruction syndrome including follow-up in a 2-year-old boy with neuroblastoma after a high dose of busulfan/melphalan and autologous stem cell transplantation. Ten days after stem cell reinfusion he developed a fever, increasing belly circumference and weight gain. Clinical and laboratory parameters initially indicated moderate sinusoidal obstruction syndrome. A few days later, clinical and imaging findings indicated severe sinusoidal obstruction syndrome. The patient was treated successfully with fluid restriction, diuretics and defibrotide. **a, b** Transverse gray-scale US images show initial gallbladder wall edema of 11 mm (**a**), which resolved 3 weeks later (**b**). **c, d** Transverse gray-scale US images in the lower abdomen show moderate ascites (**c**), which diminished on follow-up (**d**). **e** An axial intercostal US color Doppler image shows the absence of normal triphasic hepatic venous outflow pattern as a result of congestion-related loss of liver compliance. **f** An axial intercostal color Doppler US image shows how the normal hepatic vein flow pattern was restored on follow-up. **g** An axial intercostal color Doppler US image shows initial increased pulsatility of the portal vein. **h** An axial intercostal color Doppler US image shows how this improved on follow-up. **i** A transverse US color Doppler image shows collateral blood flow along the falciform ligament and in the paraumbilical veins (no follow-up image available). **j** A coronal reformatted non-contrast-enhanced three-dimensional (3-D) T1-weighted fast field echo magnetic resonance (MR) image (repetition time/echo time 5.89/0 ms) performed 4 weeks after the start of sinusoidal obstruction syndrome treatment shows slight heterogeneity of the liver (*circle*). **k** This was not visible several months earlier on coronal reformatted non-contrast-enhanced 3-D T1-weighted fast field echo MRI. **l** The heterogeneity resolved several months later on coronal reformatted non-contrast-enhanced 3-D T1-weighted fast field echo MRI. No elastography measurements were performed

Most of the described US features of sinusoidal obstruction syndrome can be derived from the increased hepatic vascular inflow resistance and hepatic congestion that result from the occlusion of hepatic sinusoids and terminal hepatic venules. Portal vein flow may be increased, normal, or show decreased respiratory velocity variations or increased pulsatility, especially in the early or subclinical stage of sinusoidal obstruction syndrome. Also, segmental (distal) portal vein flow reversal may precede clinical manifestations [25]. Later, flow velocity can decrease, show a to-and-fro pattern or ultimately reverse completely (Fig. 1). The latter two are, however, infrequently observed. Although a specific sign of sinusoidal obstruction syndrome in the appropriate clinical context, portal vein flow reversal occurs generally too late in the disease course to affect early treatment. This is why the recent international expert position statement on sinusoidal obstruction syndrome by Mahadeo et al. [5] stated against using reversed portal flow for diagnosis. Abnormal portal flow characteristics resolve over time with resolution of the clinical sinusoidal obstruction syndrome signs and symptoms. Gallbladder wall edema occurs because of the impaired venous drainage of the gallbladder into the right portal vein. Ascites and gallbladder wall edema were shown to be independent predictors in 70 children with clinically suspected sinusoidal obstruction syndrome after hematopoietic stem cell transplantation [26]*.* Another example is the absence of the characteristic triphasic hepatic venous outflow pattern as a result of congestion-related loss of liver compliance [14, 17, 19, 27]*.* Figures 2 and 3 illustrate the spectrum of US findings related to sinusoidal obstruction syndrome in a patient with nephroblastoma and in a patient with neuroblastoma. The latter includes follow-up images and correlation with MRI.

**Fig. 4:**
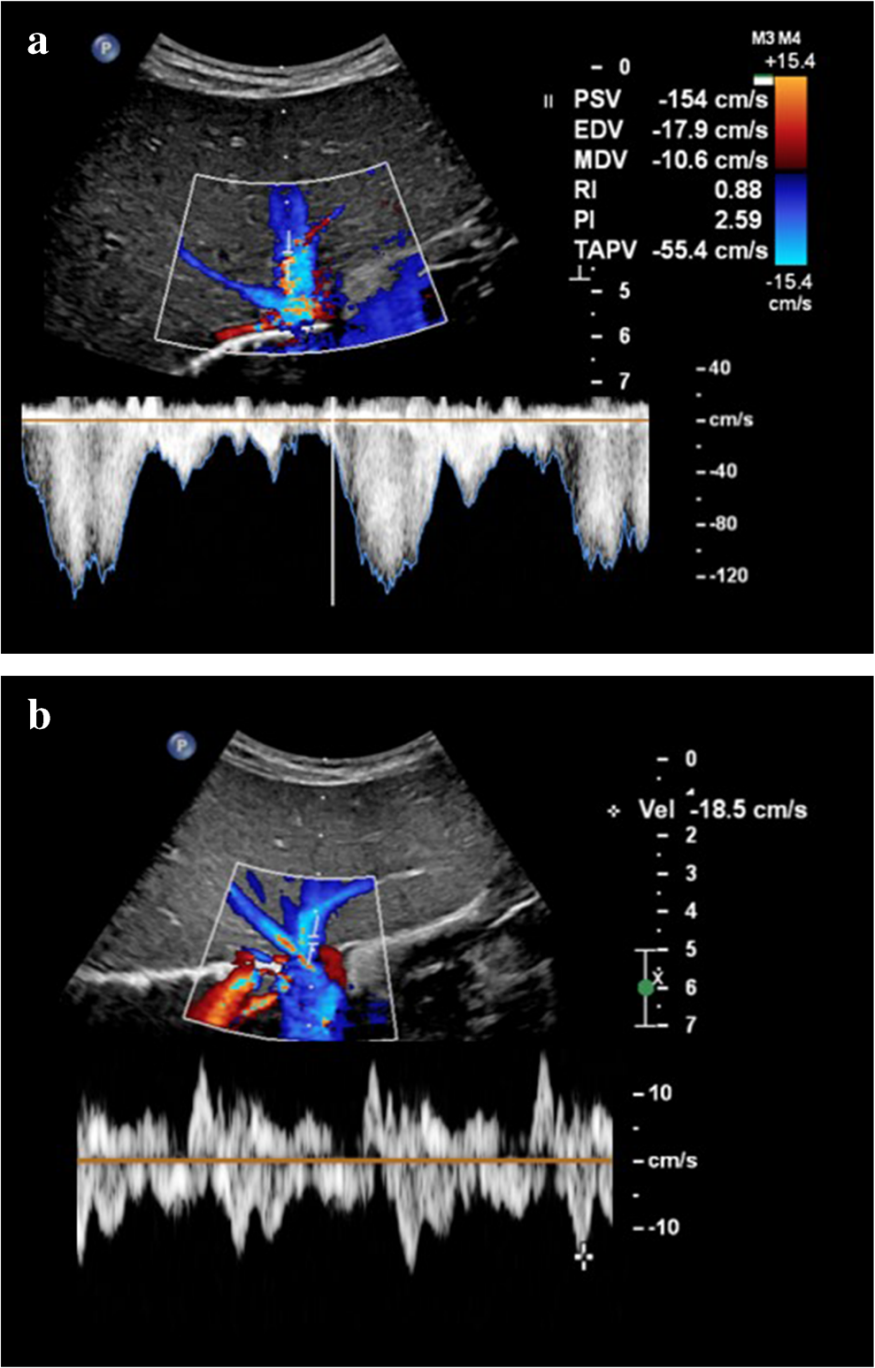
Increased hepatic vein velocities during sinusoidal obstruction syndrome in a 3-year-old with stage 4 neuroblastoma treated with high-dose busulfan/melphalan followed by autologous stem cell transplantation who developed severe sinusoidal obstruction syndrome for which defibrotide was administered. **a** An intercostal transverse ultrasound (US) image shows how hepatic vein flow reached very high peak velocities of 154 cm/s during the sinusoidal obstruction syndrome episode, with coinciding high portal vein velocities of 40 cm/s (not shown). **b** An intercostal transverse US image shows how hepatic vein velocities normalized during follow-up with peak velocities of up to 18.5 cm/s. Hepatic vein flow velocites also normalized

From our personal experience, hepatic vein peak systolic velocity can increase in the subclinical or early stages as well as during the course of sinusoidal obstruction syndrome, with peak velocities sometimes exceeding 120 cm/s and normalizing as the clinical condition improves (Fig. 4). To our knowledge, this US feature has not been studied extensively yet. Park et al. [26] found that peak velocity in the hepatic vein was somewhat higher in their veno-occlusive disease group compared with the non-veno-occlusive disease group (42.2±15.8 cm/s vs. 38.7±17.8 cm/s, respectively, *P*=0.23). Since we also encounter increased portal vein flow velocities and increased hepatic artery flow velocities in the early or subclinical course of sinusoidal obstruction syndrome, we hypothesize that an initial compensatory hemodynamic mechanism of the liver precedes clinical sinusoidal obstruction syndrome and the well-established Doppler imaging features (Table 2).

Flow in the paraumbilical veins in relation to sinusoidal obstruction syndrome was among other US parameters studied by Lassau et al. [15]. They found in a multivariate analysis performed in a group of 71 children undergoing myeloablative therapy with busulfan before hematopoietic stem cell transplantation that splenomegaly, ascites and flow recorded in the paraumbilical veins correlated with the severity of sinusoidal obstruction syndrome.

Recently, Nishida et al. [28] published a new US scoring system for sinusoidal obstruction syndrome after hematopoietic stem cell transplantation in adults*.* Their HokUS-10 scoring system included 10 US/Doppler items: hepatic left and right lobe diameter (≥70 mm and ≥110 mm, respectively), gallbladder wall thickening (≥6 mm), portal vein diameter (≥12 mm), paraumbilical vein diameter (≥2 mm) and flow signal, the amount of ascites (mild, moderate or severe), mean portal vein velocity (<10 cm/s) and flow direction (congestive or hepatofugal), and hepatic artery resistive index (>0.75). A cutoff value of 5 points for a positive diagnosis of sinusoidal obstruction syndrome yielded the highest area under the receiver operator curve (0.980), with a sensitivity of 100%, specificity of 96%, positive predictive value of 71% and negative predictive value of 100%. This scoring system is yet to be validated in the pediatric population.

## Computed tomography

**Fig. 5:**
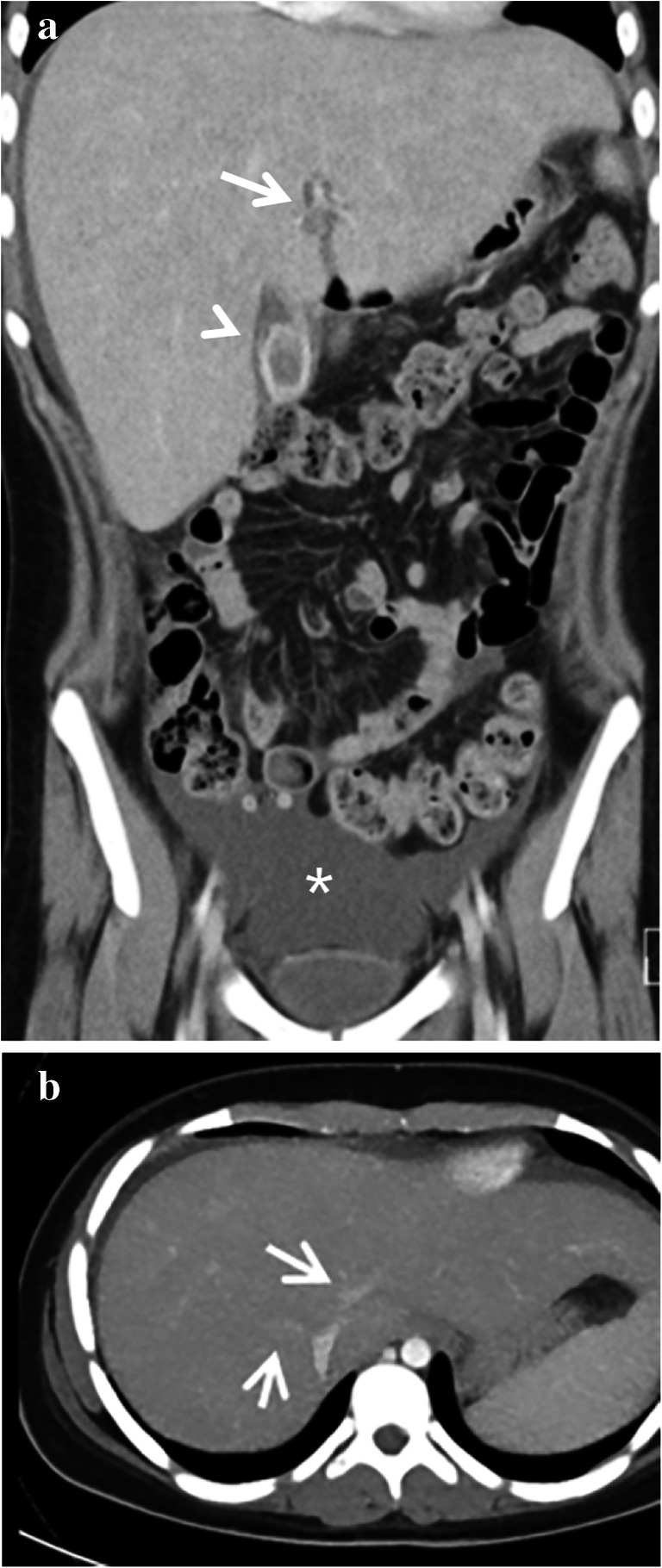
Computed tomography (CT) features of sinusoidal obstruction syndrome. A CT scan in a 14-year-old girl (same patient as in Fig. 1c) with stage 4 neuroblastoma was performed a month after high-dose busulfan/melphalan and autologous stem cell transplantation. She had a history of gallstones. She presented with nausea, right upper quadrant pain and elevated C-reactive protein. **a** A coronal contrast-enhanced abdominal CT shows enlarged liver, periportal edema (*arrow*), gallbladder wall thickening (*arrowhead*) and ascites (*asterisk*). **b** A transverse abdominal contrast-enhanced CT shows narrowing of the hepatic veins (*arrows*). The thickened gallbladder wall was initially interpreted as acute cholecystitis. However, the elevated bilirubin, elevated liver transaminases in combination with liver enlargement, ascites and a recent history of busulfan/melphalan chemotherapy and autologous stem cell transplantation were diagnostic for severe sinusoidal obstruction syndrome. This patient was treated conservatively at the time and recovered. She has not had symptomatic cholecystolithiasis or cholecystitis since

Due to radiation exposure, CT is not the imaging modality of choice for evaluating sinusoidal obstruction syndrome in children. Signs of sinusoidal obstruction syndrome can, however, be a coincidental finding when a CT is performed for a different indication. As with US, ascites, hepatomegaly, periportal edema, gallbladder wall thickening, hepatic vein narrowing, widening of the hepatic artery and the formation of collaterals can be recognized on CT. In addition, heterogeneous hypo-attenuation of liver parenchyma and patchy enhancement have been described (Fig. 5). In a recent meta-analysis, the latter two features with or without narrowing or invisible hepatic veins in the portal equilibrium phase were shown to be the most important CT features for diagnosing sinusoidal obstruction syndrome in adults [29].

Literature on CT findings of pediatric sinusoidal obstruction syndrome is scarce. A pictorial essay on US and CT findings in hepatic veno-occlusive disease was published in 1993 by Benya et al. [30], illustrating periportal edema and ascites on CT in a 15-year-old with hepatic veno-occlusive disease after bone marrow transplantation. In 2009, Khoury et al. [31] published a paper on imaging findings of abdominal complications of chemotherapy in pediatric malignancies. Here a CT image of the liver in a 1-year-old boy with neuroblastoma, abnormal liver function tests and a diagnosis of hepatic sinusoidal obstruction syndrome was shown, illustrating hepatomegaly and periportal edema [31]. To the best of our knowledge, no other publications on CT findings of pediatric sinusoidal obstruction syndrome are available.

## Magnetic resonance imaging

**Fig. 6:**
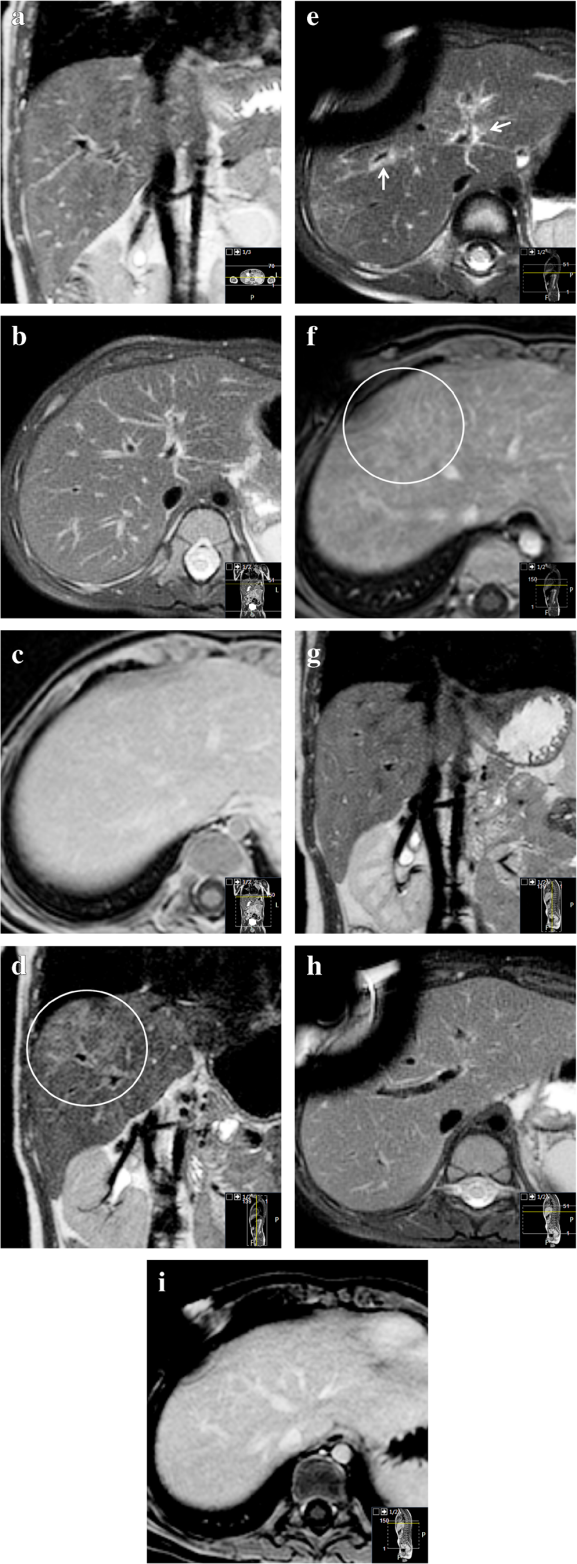
Transient chemotherapy-related hepatic abnormalities on magnetic resonance imaging (MRI) for possible subclinical sinusoidal obstruction syndrome. A 2-year-old boy with Beckwith-Wiedemann syndrome with stage-I high-risk nephroblastoma was treated according to the SIOP (Société Internationale d’Oncologie Pédiatrique/International Society of Paediatric Oncology) 2001 protocol with actinomycin D, vincristine and doxorubicin. No liver abnormalities were present on the MRI before treatment. **a–c** Pretreatment coronal three-dimensional T2-weighted turbo spin echo MRI (**a**) (repetition time [TR]/echo time [TE] 447/90 ms), transverse T2-weighted fat-saturated multivane xd MRI (**b**) (TR/TE 2,666/67.1 ms) and gadolinium-enhanced transverse T1-weighted high-resolution isotropic volume examination MRI (**c**) (TR/TE 5.46/2.68 ms) show no abnormalities of the liver. **d–f** Eight months later, during the seventh month of postoperative treatment, identical MRI sequences at the same anatomical locations show hepatic abnormalities. The images show patchy T2 hyperintensities (*circle* in **d**), periportal edema (*arrows*) and a reticular enhancement pattern (*circle* in **f**). There was no liver enlargement, no gallbladder edema and no relevant ascites. Clinically, there were no signs or symptoms of sinusoidal obstruction syndrome. Liver enzymes were not tested at that time. Platelet count was low (53×10^9^/L (normal 150–450×10^9^/L); in combination with leukopenia, this was considered to be caused by the chemotherapy. The differential is a subclinical sinusoidal obstruction syndrome [8]. **g–i** Follow-up MRI another 7 months, again with identical sequences, planes and anatomical location to (**a**), (**b**) and (**c**), show that the abnormalities spontaneously resolved while the patient was still on treatment. Susceptibility artefacts originate from the permanent intravenous catheter

As with CT, available literature of MRI features of sinusoidal obstruction syndrome focuses on adults. We found no papers describing pediatric cases. Similar to findings on US and CT, ascites, hepatomegaly, periportal edema, and gallbladder wall thickening are general features that can be recognized on MRI [14]. From our experience, incidental transient patchy hepatic T2 heterogeneities with reticular enhancement with or without ascites can be encountered on follow-up MRI scans during the treatment of nephroblastoma patients on therapy including vincristine and actinomycin D (Fig. 6). Generally, these patients will have no clinical symptoms of sinusoidal obstruction syndrome, although thrombocytopenia often seems to coincide with these incidental MRI abnormalities. As shown by Jagt et al. [8], almost half of nephroblastoma patients pretreated with regimens containing actinomycin D have histological proof of sinusoidal obstruction syndrome on liver biopsies during tumor resection, whereas clinical manifestations of sinusoidal obstruction syndrome were present in a minority of these patients. Therefore, we hypothesize that these hepatic abnormalities on MRI could be manifestations of subclinical sinusoidal obstruction syndrome. From our experience, these subclinical liver MRI abnormalities usually resolve spontaneously on subsequent follow-up MRI (Fig. 6).

## Follow-up

**Fig. 7:**
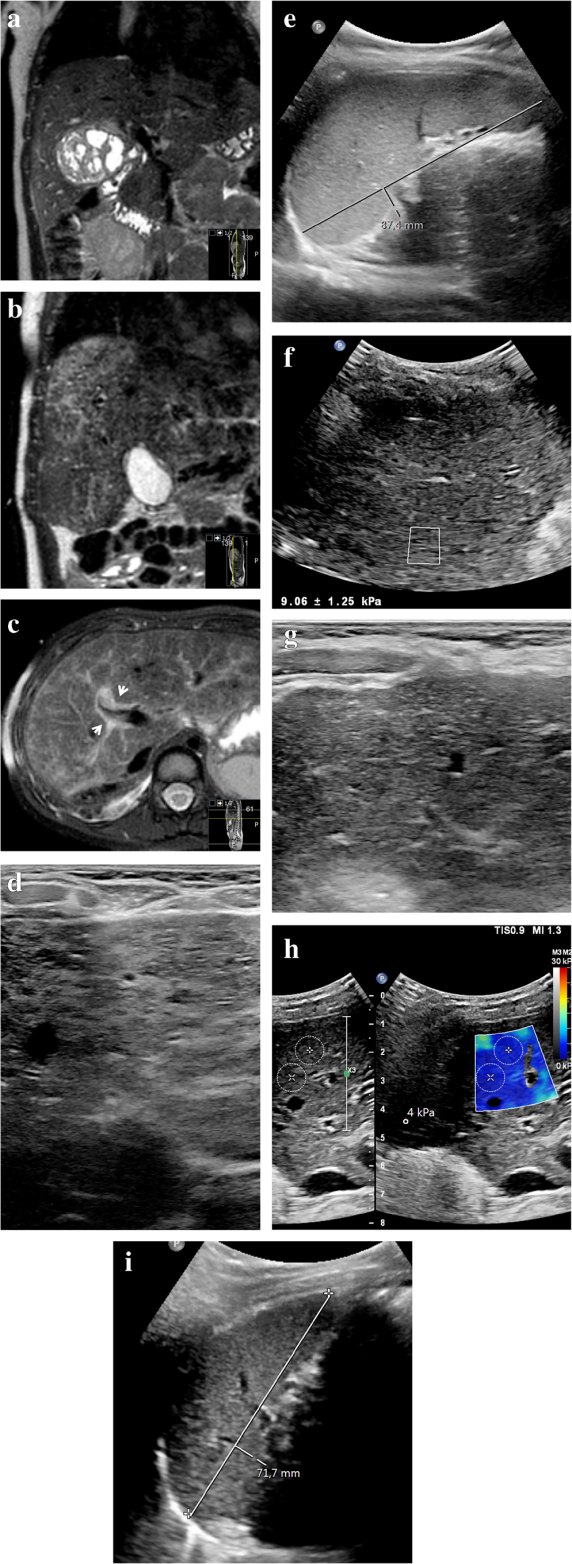
Long-term liver abnormalities in a 3-year-old girl with stage 2 nephroblastoma, intermediate risk. **a** Coronal three-dimensional (3-D) T2-weighted turbo spin echo magnetic resonance (MR) image (repetition time [TR]/echo time [TE] 447/90 ms) shows the right renal tumor mass before treatment. An intraoperative liver sample showed confluent areas of acidophilic degenerated hepatocytes in the proximity of the central hepatic veins, a finding that could be in keeping with sinusoidal obstruction syndrome or chemotherapy-related toxic damage. **b, c** Follow-up imaging at the end of the postoperative treatment regimen with actinomycin D and vincristine. Coronal 3-D T2-weighted turbo spin echo MRI (**b**) (TR/TE 445/90 ms) and transverse T2-weighted fat-saturated multivane xd MRI (**c**) (TR/TE 3,294/67.1 ms) show enlargement of the liver from baseline (**c**), with diffuse patchy heterogeneous T2 hyperintensities and periportal edema (*arrows*). **d** A transverse high-resolution ultrasound (US) image (linear 12–5 MHz probe) of the liver shows heterogeneous liver parenchyma in keeping with the MRI. **e** A sagittal grey-scale US image shows an enlarged spleen of 8.7 cm. **f** An elastography measurement of the liver parenchyma via intercostal approach shows an elevated stiffness of 7.3 kPa (Epiq ElastPQ, Philips Healthcare, Best, The Netherlands). The patient had not experienced clinical signs of sinusoidal obstruction syndrome other than mildly elevated liver transaminases, with normal bilirubin. Hepatomegaly in combination with minimal free fluid (not shown) matches mild sinusoidal obstruction syndrome. No specific treatment for sinusoidal obstruction syndrome was given. **g** The liver parenchyma remains heterogeneous on follow-up US 2 years later (transverse high-resolution US image of the liver). **h** The elastography measurement (transverse image) of the liver normalizes to 4 kPa (Philips Epiq ElastQ) on the same follow-up US. **i** A sagittal grey-scale US image at follow-up shows that the size of the spleen has normalized

While most imaging abnormalities related to sinusoidal obstruction syndrome in children resolve spontaneously, some do not. We have summarized the multi-modality imaging spectrum of sinusoidal obstruction syndrome over time in Table 3. To illustrate the course of liver abnormalities, Fig. 7 shows follow-up images in a 3-year-old girl with stage 2 nephroblastoma, intermediate risk. An intraoperative liver sample in this patient showed confluent areas of acidophilic degenerated hepatocytes in the proximity of the central hepatic veins, a finding that could be in keeping with sinusoidal obstruction syndrome or chemotherapy-related toxic damage. After completing postoperative treatment with actinomycin D and vincristine, follow-up MRI and US showed enlargement of the liver and spleen with periportal edema, patchy heterogeneous liver parenchyma, minimal ascites and elevated liver stiffness — findings that could well be in keeping with experienced or ongoing mild sinusoidal obstruction syndrome, although there were no clinical signs or symptoms other than mildly elevated liver transaminases. On follow-up US imaging, all abnormalities normalized except for the heterogeneous liver parenchyma, which still persists after 2 years.

**Table 3:** Summary of imaging features of sinusoidal obstruction syndrome/veno-occlusive disease (SOD/VOD) over time

	Preclinical predictors	Acute (days)	Subacute (weeks)	Longer term (months–years)
US	*Doppler:* Increased velocities in portal vein, hepatic artery and/or hepatic veins can precede clinical symptoms of SOS/VOD	*Doppler:* Portal vein: -Usually a normal flow direction -Increased pulsatility, decreased respiratory variations -(Segmental) flow reversal possible -Increased, normal or decreased velocity Increased peak systolic velocity and elavated resistive index of hepatic artery Hepatic veins: -Increased, normal or decreased peak systolic velocities -Loss of triphasic flow pattern Paraumbilical vein collaterals present	*Doppler:* -Complete spectrum of portal vein flow abnormalities can be seen, including reversed flow direction -Gradual normalizing of elevated velocity and restrictive index of the hepatic artery -Gradual normalizing of flow velocities and flow pattern -Paraumbilical vein collaterals diminish	*Doppler:* Normalizes
	*B-mode:* Gradual onset of: -hepatomegaly -gallbladder wall edema -periportal edema -ascites -congested heterogeneous appearance of liver parenchyma	*B-mode:* -Hepato(spleno)megaly -Gallbladder wall edema -Periportal edema -Ascites -Hepatic vein narrowing *-*Visualization of collaterals -Congested heterogeneous appearance of liver parenchyma	*B-mode:* -Hepato(spleno)megaly -Gallbladder wall edema -Periportal edema -Hepatic artery hypertrophy -Ascites -Hepatic vein narrowing -Visualization of collaterals -Heterogeneous liver parenchyma	*B-mode:* -Heterogeneous liver parenchyma can persist -Other features normalize
	*Elastography:* Increase from baseline	*Elastography:* Increase from baseline	*Elastography:* Increases	*Elastography:* Decreases/normalizes
CT		-Hepato(spleno)megaly -Gallbladder wall edema -Periportal edema -Hepatic vein narrowing -Hepatic artery hypertrophy -Ascites -Heterogeneous parenchyma attenuation pattern		-Heterogeneous liver parenchyma can persist -Other features normalize
MRI		-Hepato(spleno)megaly -Gallbladder wall edema -Periportal edema -Ascites -T2 or post-contrast parenchymal heterogeneities		-Heterogeneous liver parenchyma can persist -Other features normalize

## Recommendations

Although abnormalities related to sinusoidal obstruction syndrome can be recognized on several imaging modalities, US is the technique of choice in children. Combining B-mode- Doppler- and elastography-derived information can aid in early detection, treatment evaluation and follow-up. Also, alternative diagnoses can be suggested. A baseline US examination before the start of treatment in children at risk of developing sinusoidal obstruction syndrome can facilitate the detection of related changes. To this purpose, Mahadeo et al. [5] have recently published radiology reporting templates to aid in structured US evaluation. As stated, hepatic parenchymal abnormalities can remain visible on imaging for years after sinusoidal obstruction syndrome. Although much is still unknown about the long-term effects of childhood sinusoidal obstruction syndrome, long-term complications are rare, and no intensive (imaging) follow-up scheme is deemed necessary.

## Conclusion

Imaging features that may confirm or diminish the clinical suspicion of hepatic sinusoidal obstruction syndrome in children are very helpful to clinicians. Therefore, a thorough understanding of the possible manifestations of sinusoidal obstruction syndrome on different imaging modalities, including long-term abnormalities, is crucial to the (pediatric) radiologist. In addition, a baseline and follow-up liver US examination including elastography in children at risk of developing sinusoidal obstruction syndrome can help diagnose the condition in an early stage for a more favorable clinical outcome.
